# 

**Published:** 1875-11

**Authors:** 


### Table of Contents.

ORIGINAL COMMUNICATIONS.

Treatment of Uterine Displacements by Genu-Pectoral Posture and Pneumatic Pressure. *By T. S. Hopkins, M.D., of Thomasville, Ga.* 449
Bromide of Potassium and Quinia Combined, in the Treatment of Intermittent Fever. *By T. S. Dekle, M.D., of Thomas county, Ga.* 452
Mania a Potu. *By L. A. Dugas, M.D., of Augusta* 460
Report of two Cases of Lithotomy. *By E. A. Jelks, M.D., of Quitman, Brooks county, Ga* 462
Report of a Case of Incised Wound of the Upper Lid and Eye. *By Richard M. Lewis, M.D., of Savannah, Ga* 464
Treament of Diphtheria. *By Lawrence Alexander, M.D., of Yorkville, South Carolina* 466
Report of Miscellaneous Cases Occurring Since the Last Meeting of the Georgia Medical Society, Savannah, Ga. *By Henry LeHardy, M.D., Reporter* 468
Clinic Atlanta Medical College. *By A. W. Calhoun, M.D., Professor of Diseases of the Eye and Ear* 471
Atlanta Accademy of Medicine 474

OUR EXCHANGES.

Battey's Operation 481
The Climate of Florida 492
On the Use of Chloral Hydrate 494
Treatment of Cerebral Rheumatism by Hydrate of Chloral 495
Membranous Croup 496
Croup and Diptheria 497
On Meat and Milk 498
On Anthelmintics 499
Discharge of an Ovum and its Relation in Point of Time to Menstruation 500
Women in Medicine, Law and Theology 501
A Case of Traumatic Ventral Hernia 502
The Use of Revulsives in Diseases of the Nervous System 503
A New Medical School in Glasgow 404

BIBLIOGRAPHICAL.

Human Physiology 505
Lectures on Syphilis—A Manual of Minor Surgery and Bandaging 506
Vision, its Optical Defects and Adaptation of Spectacles—Physicians' Visiting List List—Transactions of the Medical Society of the District of Columbia—What Course Should be Pursued with an Eye Lost Through Accident—Two Cases of Exophthalmic Goitre, Associated with Chronic Uticaria—The Relations of the Urine to Diseases of the Skin—American Public Health Association—Wanted for the Surgeon General's Library 507

EDITORIAL.

New Device for Epistaxis—The Physicians of Oldham county, Ky 508
The South Georgia Medical Association 511
Medical Association of Georgia 512

